# GAM-SpCaNet: Gradient awareness minimization-based spinal convolution attention network for brain tumor classification

**DOI:** 10.1016/j.jksuci.2023.01.002

**Published:** 2023-02

**Authors:** Chaosheng Tang, Bin Li, Junding Sun, Shui-Hua Wang, Yu-Dong Zhang

**Affiliations:** a School of Computer Science and Technology, Henan Polytechnic University, Jiaozuo, Henan 454000, PR China; b School of Computing and Mathematical Sciences, University of Leicester, Leicester LE1 7RH, UK; c Department of Information Systems, Faculty of Computing and Information Technology, King Abdulaziz University, Jeddah 21589, Saudi Arabia

**Keywords:** Brain tumor classification, Gradient awareness minimization, Intermittent fully connected layer, Positional attention convolution block, Relative self-attention transformer block

## Abstract

Brain tumor is one of the common diseases of the central nervous system, with high morbidity and mortality. Due to the wide range of brain tumor types and pathological types, the same type is divided into different subgrades. The imaging manifestations are complex, making clinical diagnosis and treatment difficult. In this paper, we construct SpCaNet (Spinal Convolution Attention Network) to effectively utilize the pathological features of brain tumors, consisting of a Positional Attention (PA) convolution block, Relative self-attention transformer block, and Intermittent fully connected (IFC) layer. Our method is more lightweight and efficient in recognition of brain tumors. Compared with the SOTA model, the number of parameters is reduced by more than three times. In addition, we propose the gradient awareness minimization (GAM) algorithm to solve the problem of insufficient generalization ability of the traditional Stochastic Gradient Descent (SGD) method and use it to train the SpCaNet model. Compared with SGD, GAM achieves better classification performance. According to the experimental results, our method has achieved the highest accuracy of 99.28%, and the proposed method performs well in classifying brain tumors.

## 1 Introduction

Brain tumors are abnormal tissues in the brain that consist of cancer cells that can continuously differentiate. It will compress nerve tissue and cause great pain to the patient, such as headaches, weakness, numbness, nausea, vomiting, or seizures. The survey shows that the average cost of treating a brain tumor patient is $1.9 million in the United States, which places a huge financial burden on families and society. The World Health Organization divides brain tumors into four grades. The higher the tumor grade, the lower the prognosis and survival rate (Addeh and Iri, 2021). That is, an early cancer diagnosis can detect potential tumors and prevent them from developing further and deteriorating into cancer. Non-invasive methods, such as CT and MRI, diagnose most brain tumors. However, the manual evaluation of brain tumor images is complicated, and the current non-invasive diagnosis requires rich clinical experience, which quickly causes misdiagnose (Afshar et al., 2018).

Deep learning techniques provide great advantages for medical image analysis and can better diagnose brain tumors. Zhou et al. (Zhou, 2018) trained images of different types of brain tumors by recurrent neural networks and got 92.13 % accuracy via DenseNet-LSTM. The key to his research is to directly use the entire sequence of 3D images as training samples to model slices of 2D images, omitting the time-consuming process of individually labeling each frame in the sequence. Chang et al. (Chang, 2017) conducted a retrospective study of MR imaging data and molecular data of 259 patients with gliomas. They proposed 2D/3D hybrid CNN to classify IDH1 mutation and 1p19q co-deletion. Different from previous research, the principal component analysis technique was employed to determine the most predictive imaging features of each molecular state. The prediction accuracy rates are 94 % and 92 %, respectively. Yang et al. (Yang et al., 2018) analyzed the performance of AlexNet and GoogLeNet in differentiating gliomas. They compared the accuracies of the two CNNs trained from scratch with pre-trained CNN. The results show that a pretrained CNN and GoogLeNet can achieve 94.5 % accuracy. Jiang et al. (Linqi and Jingyang, 2022) proposed SE-ResNeXt to simplify the classification process of gliomas. Three optimization methods are used in their study. Firstly, a multi-step learning strategy is used to adjust the learning rate dynamically. Second, the label smoothing strategy is adopted to optimize the unique thermal labels to reduce the network's reliance on the true label probability distribution and improve the network's prediction ability. Finally, transfer learning method based on the CE-MRI simplifies migration learning process. The accuracy and specificity reach 98.99 % and 98.33 % on the BraTS2019 dataset, respectively. Gull et al. (Gull and Khan, 2021) proposed the fully convolutional neural network (FCNN) and transfer learning techniques for brain tumor detection. The framework is divided into five stages: preprocessing, skull dissection, tumor segmentation, post-processing, and binary classification. In addition, the global thresholding technique has been employed to eliminate the enhanced small non-tumor regions, and a focal loss function is used to solve the problem of category imbalance. The average classification accuracy is 96.49 %, 97.31 % and 98.79 %, respectively. Rao et al. (Rao, 2022) proposed a kernel support vector machine (KSVM) and social ski driver (SSD) algorithm in the study of tumor classification. They used NMF-based preprocessing to perform image smoothing and quality enhancement and divided the image into non-overlapping regions by the binomial thresholding segmentation method. During the preprocessing of classification, they combined GLCM with SGLDM to handle feature extraction, which can be used to select the best subset of features by a meta-heuristic HHO algorithm.

However, few studies have reported on the following three issues. First, the position information of the feature map will be lost during the feature extraction process, which results in insufficient feature extraction. As the number of convolutional layers increases, the perceptual field of the feature map mapped to the original image becomes larger, and the perception of position information becomes poorer, thus losing a certain amount of position information. This leads to the underutilization of position information. Amirul et al. (Islam and Bruce, 2001) explain how position information is exposed to neural network learning. Experiments show that position information is implicit in the extracted feature maps and can be utilized to a large extent. However, recent research rarely takes position information into account in the diagnosis of brain tumors.

Second, sharpness-based learning methods suffer from the rescaling sensitivity of model parameters, which weakens the correlation between sharpness and the generalization gap. Recently, many scholars have studied the generalization of the deep neural network to solve the shortcomings of pure optimization. They have attempted to elucidate the relationship between the geometry of the loss surface and generalization performance, where the minimization of the sharpness of the loss surface and the derived generalization boundary has been proven effective (Sun et al., 2021; Chaudhari, 2019/12/20 2019.; M. H, 2016; Hochreiter and Schmidhuber, 1997). However, even sharpness-based learning methods, including SAM (Foret et al, 2010) and some sharpness measures, will be sensitive to rescaling model parameters. Dinh et al. (Dinh et al., 2017) pointed out that parameter rescaling without changing the loss function leads to differences in sharpness values, so that this feature may weaken the correlation between sharpness and generalization error. To compensate for the scale-dependent sharpness problem, scholars have conducted many studies recently (Tsuzuku et al., 2020; Yi et al, 2019; Liang et al., 2019; Karakida and Amari, 1906). However, these previous works are limited to proposing generalization measures that do not suffer from scale-dependent problems.

Third, redundant parameters and overfitting problems are caused by the fully connected (FC) layer. Because of fully connected characteristics, FC generally has the most parameters. Traditionally, as the last layer of the model, FC acts as a classifier. However, the size of FC weight rises dramatically with the increase of network scale, which quickly causes overfitting. Although current research (Byerly and Dear, 2021; Kowsari et al., 2018; Bengio et al., Aug 2013; Ciregan et al., 2012) has focused on this problem, its efficiency and poor recognition accuracy have not been well addressed.

To solve the above problems, we propose a new computer-aided diagnosis method for diagnosing suspected brain tumors. The main contributions are as follows:

1) Reinforced attention (RA) is proposed to preserve long-range spatial dependencies and precise position information to enhance the attention of objects.
2) To solve the problem of scale dependence and improve the generalization ability, we design a GAM optimization algorithm.
3) To prevent the loss of essential features and solve the problems of parameter redundancy and overfitting, we propose an IFC layer.

The rest structure is as follows: Section 2 shows the dataset and preprocessing process, Section 3 describes the methodology, Section 4 shows the experimental result and discussion, and Section 5 provides a conclusion and subsequent work.

## 2 Data preprocessing

### 2.1 Source of dataset

**Fig. 1:**
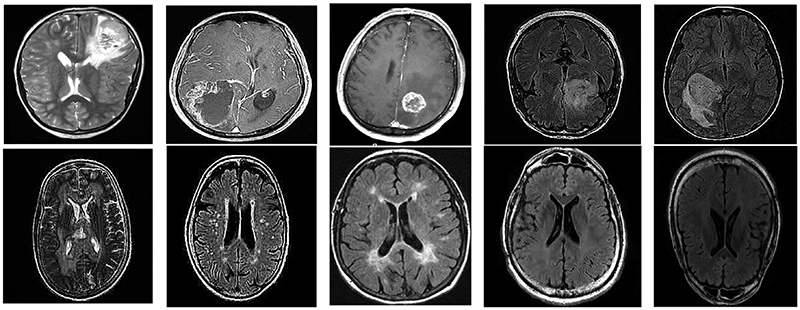
Dataset in this study.

To make the experimental process easier to implement and the experimental results more comparable, we used the BraTS2019 dataset that has a total of 3040 images containing brain tumor patient MRI and benign patient MRI. Fig. 1 shows a sample dataset of this paper. Among them, the first row is malignant patients, and the second is benign patients. We randomly select 80 % of each class of images as the training set. The remaining 20 % of images are used as the test set. We keep the same division ratio of the training set and test set to perform 5-fold cross-validation.

### 2.2 Data augmentation

**Fig. 2:**
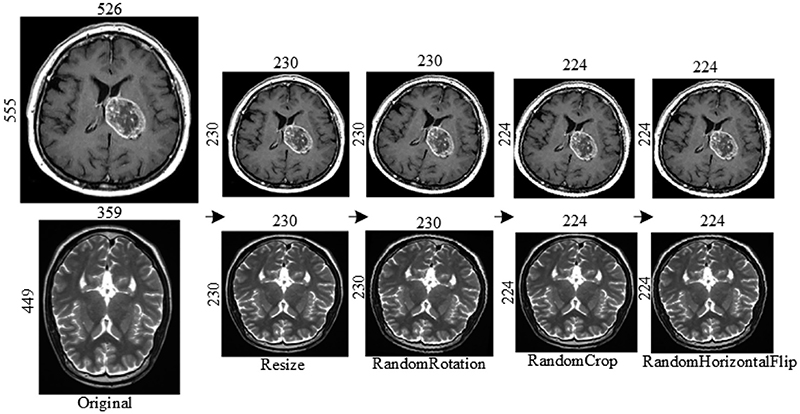
Data augmentation process.

Due to the dataset in this study coming from different sources and the size of images varying wildly, we have performed different data augmentation operations on the BraTS2019 dataset, including resize, random rotation, random crop, and random horizontal flip. The specific operation is shown in Fig. 2, which has an example of two images. First, considering the different sizes of each sample image, all images are resized to 230 x 230, then we randomly rotate them by 15 degrees. Finally, we randomly crop the images to 224 x 224 and perform random horizontal flip operations. By data augmentation operation, the robustness of the network model can be effectively improved (Perez, 2017).

## 3 Methodology

### 3.1 SpCaNet

Although transformers have larger capacities, they may have poorer generalization ability than convolutional neural networks (CNNs) (Wu, 2021). We devise a tandem stacking approach to integrate inductive biases of convolution into the transformer by (a) imposing local perceptual fields for the attention layer and (b) adding attention and feedforward neural network layers with implicit or explicit convolution operation.

Depth-wise convolution and self-attention are the sums of weighted values for each dimension in the predefined receptive field. Convolution relies on a fixed kernel to collect information about the local receptive field:

(1) $${\text{Z}}_{\text{i}}=\sum_{\text{j}\in \zeta (\text{i})}{\text{W}}_{\text{ij}}⊙{\text{x}}_{\text{j}}.$$

In Eq. (1), X_i_, Z_i_, ∈ R^D^ are the input and output at position i respectively, w is weight matrix and ζ_i_ is local neighborhood in position i. Depth-wise convolution has translation invariant characteristics. The convolution weight w_ij_ focuses on the relative bias offset between i and j, instead of specific value of i and j. The translation invariance has improved the generalization capability.

In contrast, the perceptual field of self-attention (Vaswani, et al., 2017) is not a local neighborhood, and its weights are calculated based on pairwise similarity and then activated by the softmax function. As shown in Eq. (2):

(2) $$z_{i}=\sum_{j\in G}{\frac{\text{exp}\;(x_{i}^{T}x_{j})}{\underset{\;}{\underset{︸}{\sum_{k\in G}\text{exp}\;(x_{i}^{T}x_{k})}}}}_{A_{ij}},$$

where G represents the global space, A_ij_ represents attention weight and x_i_, x_j_ are two patches of an image. The input of self-attention is adaptively weighted. This makes self-attention easier to capture relationships on different elements. Besides, self-attention provides a global receptive field, which can obtain more contextual information than CNN's local receptive field.

As is shown in Eq. (3) and Eq. (4), to merge the input of self-Attention with adaptive weighting, a global static convolution kernel is added to the adaptive attention matrix around softmax normalization, which has combined the global perceptual field with the translational invariance.

(3) $${\text{z}}_{\text{i}}^{\text{post}\;}=\sum_{\text{j}\in \text{G}}\left(\frac{\text{exp}({\text{x}}_{\text{i}}^{\text{T}}{\text{x}}_{\text{j}})}{\sum_{\text{k}\in \text{G}}\text{exp}({\text{x}}_{\text{i}}^{\text{T}}{\text{x}}_{\text{k}})}{\text{W}}_{\text{ij}}\right){\text{x}}_{\text{j}}$$

(4) $$z_{\text{i}}^{\text{pre}\;}=\sum_{\text{j}\in \text{G}}\left(\frac{\text{exp}({\text{x}}_{\text{i}}^{\text{T}}{\text{x}}_{\text{j}}+{\text{w}}_{\text{ij}})}{\sum_{\text{k}\in \text{G}}\text{exp}({\text{x}}_{\text{i}}^{\text{T}}{\text{x}}_{\text{k}}+{\text{w}}_{\text{ik}})}\right){\text{x}}_{\text{j}}$$

The global context has a quadratic complexity in terms of spatial size (Guo et al, 2022). Therefore, if relative attention is directly applied in Eq. (3) or Eq. (4) to the original input, the calculation speed will drop sharply because of the large number of pixels.

Therefore, down-sampling is adopted to lessen spatial size after the feature map reaches a manageable level and apply globalrelative attention.

**Fig. 3:**
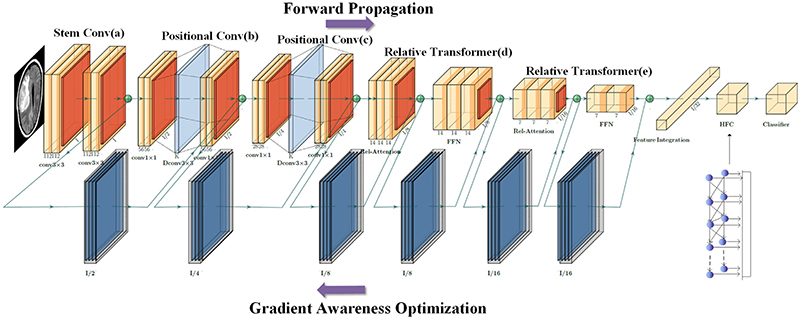
The Structure of SpCaNet.

Fig. 3 shows the general structure of SpCaNet. The stem convolution consists of two 3 x 3 convolutions designed to reduce dimensionality and make global attention feasible when the overall size increases. Compared to models using a local attention mechanism, SpCaNet always uses sufficient attention to guarantee the model's capacity.

The relative transformer, shown in Fig. 3(e), takes up most of the calculations and parameters. For all general convolution and PA convolution blocks, kernel size is set to 3. For all transformer blocks, the attention head is respectively set to 32. The inflation rate of an inverted bottleneck is 4. SpCaNet stacks convolutional and attention layers vertically. In the last layer, we adopted IFC to reduce calculation and integrate features through progressive input and feature splicing. SpCaNet gives more global information to brain tumor images, which is more sensitive to the lesion area and has the advantage of low computational overhead.

### 3.2 Reinforced attention

Positional information is crucial to generate spatial selective attentional maps. To solve the underutilization of positional information, scholars have sought to determine this problem. SENet (Hu et al., 2017) simply squeezes each 2-dimensional feature map and then constructs interdependencies between channels effectively. CBAM (Woo, et al., 2018) introduced spatial information through large-scale kernel convolution. GENet (Hu et al., 2017), GALA (Linsley et al., 2019), AA (Bello et al., 2019), and TA (Misra et al., 2021) extended the above idea by designing spatial attention and attention block.

However, SENet (Hu et al., 2017) only considers inter-channel information. CBAM and later methods mainly used convolution to capture attention information, which is insufficient to model long-term dependencies. For the optimization of the above problems, a non-local/self-attention network is built to focus on spatial and channel attention, such as GCNet (Cao et al., 2019), SCNet (Liu et al., 2020), CCNet (Huang, et al., 2019), NLNet (Wang et al., 2018), which could capture different spatial information by utilizing the non-local mechanism. However, these methods are computationally expensive. Unlike the non-local/self-attention approach, we propose a novel RA method to effectively capture position information and inter-channel relationships to enhance feature representation.

**Fig. 4:**
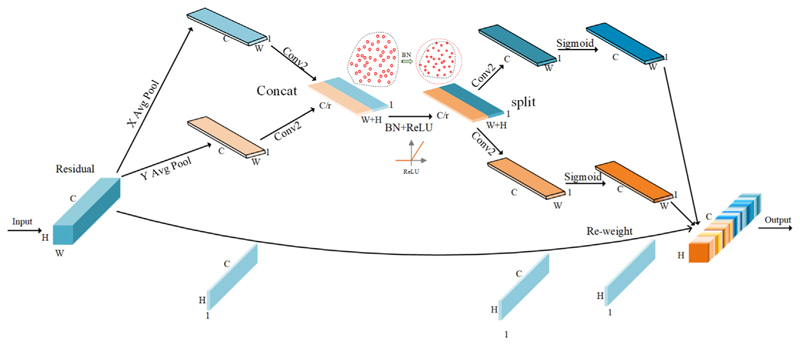
Structure of RA Block.

Fig. 4 illustrates the detailed steps of RA. Since the global pooling method compresses the global spatial information into channel descriptors, it leads to difficulty in preserving the positional information. To obtain attention to the image width and height and encode the exact positional information, the RA mechanism first divides the input feature map into two directions, width, and height. Then it performs global average pooling to obtain the feature maps in both width and height directions, respectively.

As is shown in Eq. (5) and Eq. (6), where ${\text{y}}_{\text{c}}^{\text{h}}(\text{h})$, ${\text{y}}_{\text{c}}^{\text{w}}(\text{w})$ are the output of the c-th channel at height h and width w, which is obtained by encoding each channel along horizontal and vertical coordinates using pooling kernels of size H x 1 and 1 x W.

(5) $${\text{y}}_{\text{c}}^{\text{h}}(\text{h})=\frac{1}{\;\text{W}}\sum_{0<\text{i}\leq \text{W}}{\text{x}}_{\text{c}}(\text{h},\text{i}),$$

(6) $${\text{y}}_{\text{c}}^{\text{w}}(\text{w})=\frac{1}{\text{H}}\sum_{0<\text{j}\leq \text{H}}{\text{x}}_{\text{c}}(\text{j},\text{w}).$$

Then, the width and height of the global perceptual field are stitched together and fed into a shared convolution module with a 1 x 1 convolution kernel to reduce its dimension to the original C/r. Then, the batch normalized feature map is fed into the ReLU activation function to obtain a feature map shaped as 1 x (W + H) x C/r. As shown in Eq. (7), where T1 is the 1 x 1 convolution, y^h^, y^w^ are the feature map in horizontal and vertical directions.

(7) $$\text{a}=\text{ReLU}({\text{T}}_{1}([{\text{y}}^{\text{h}},{\text{y}}^{\text{w}}])).$$

Then the feature map a ∈ R^C/rx(H+W)^ is convolved with a kernel of 1 x 1 according to the original height and width to obtain the feature map with the same number of channels as the original one, and the attention weights k^h^ and k^w^ in the height and width directions of the feature map are obtained after the Sigmoid activation function. As shown in Eq. (8) and Eq. (9), where a^h^ and a^w^ are two independent tensors obtained by splitting a in the spatial dimension, and σ is the Sigmoid function, T_h_ and T_w_ are two 1 x 1 convolutions to transform feature maps a^h^ and a^w^ to the same channels.

(8) $${\text{k}}^{\text{h}}=\sigma ({\text{T}}_{\text{h}}({\text{a}}^{\text{h}})),$$

(9) $${\text{k}}^{\text{w}}=\sigma ({\text{T}}_{\text{w}}({\text{a}}^{\text{w}})).$$

After the above calculation, the attention weights of the input feature map in the height direction and the attention weights in the width direction will be obtained. Finally, multiplying and weighting the original feature map, the final feature map with attention weights in the width and height directions will be obtained, as shown in Eq. (10).

(10) $${\text{y}}_{\text{c}}(\text{i},\text{j})={\text{x}}_{\text{c}}(\text{i},\text{j})\times {\text{k}}_{\text{c}}^{\text{h}}(\text{i})\times {\text{k}}_{\text{c}}^{\text{w}}(\text{j}).$$

### 3.3 PA convolution block

**Fig. 5:**
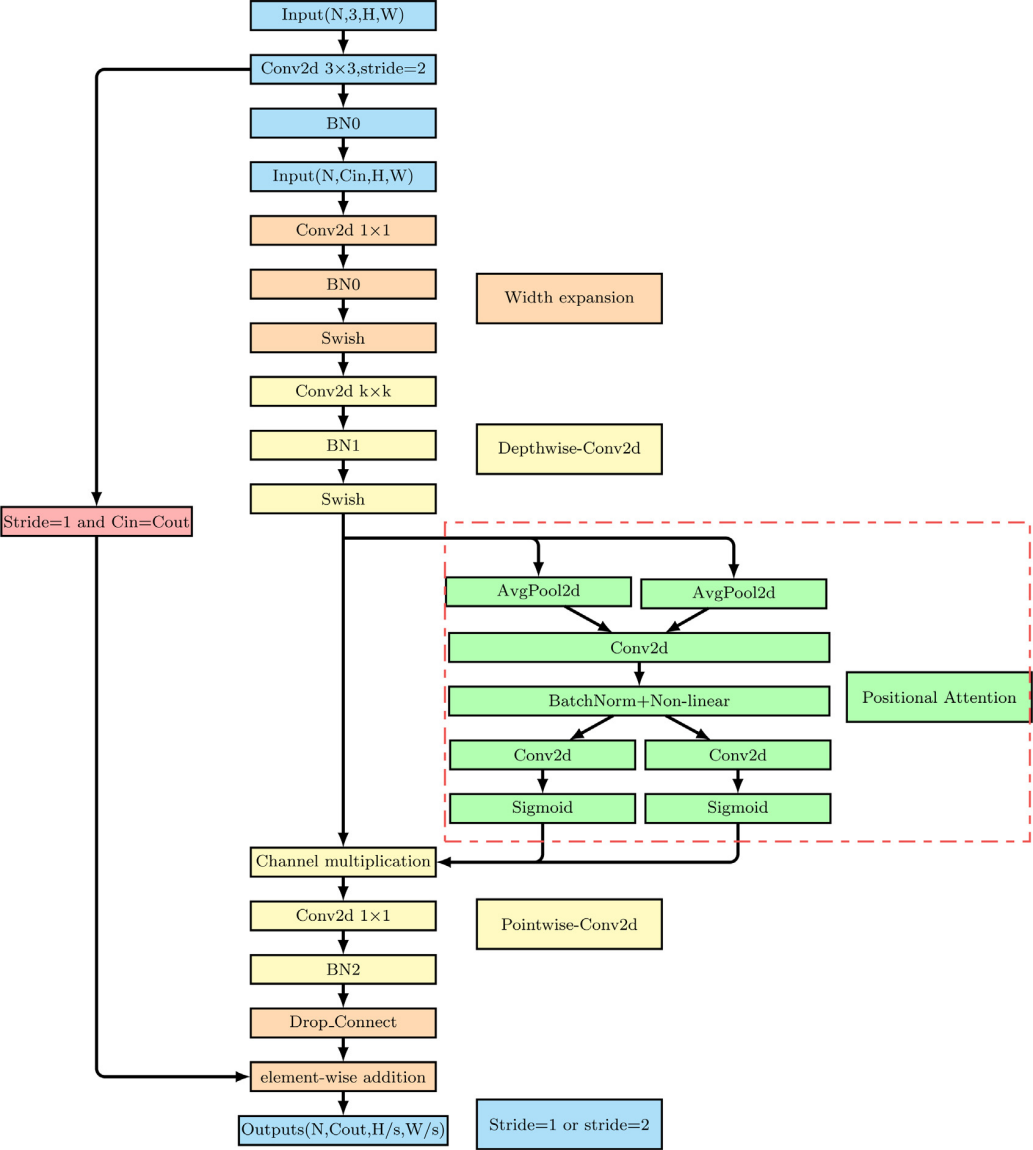
The structure of the Improved PA Convolution block.

To solve the mismatch problem of the combination of convolution and transformer, we propose PA convolution block. The overall architecture of the PA convolution block is shown in Fig. 5, which uses depth-wise convolution with inverted residuals. The expansion compression scheme is the same as the transformer's feedforward neural network module.

The PA Convolution block first performs 1 x 1 Conv for dimensionality upscaling and then performs depth-wise separable convolution. In the short connection part on the right, RA is added. First, the feature map passed from the Swish activation function is subjected to one-dimensional average pooling from x and y directions to obtain two directional feature maps with a global perception field. The feature maps obtained are stitched together, then fed into a shared 1 x 1 convolution, batch normalized, and finally passed to the sigmoid activation function. Then, channel multiplication is performed on the feature maps with the processed image by Swish (Ramachandran and Le, 2017). At last, 1 x 1 convolution is employed to reduce the feature map's dimension. After a series of batch normalization and drop-connect operations, elementwise addition is performed on the information of the short connection part on the left and the backbone of the PA convolution block to obtain the output.

**Fig. 6:**
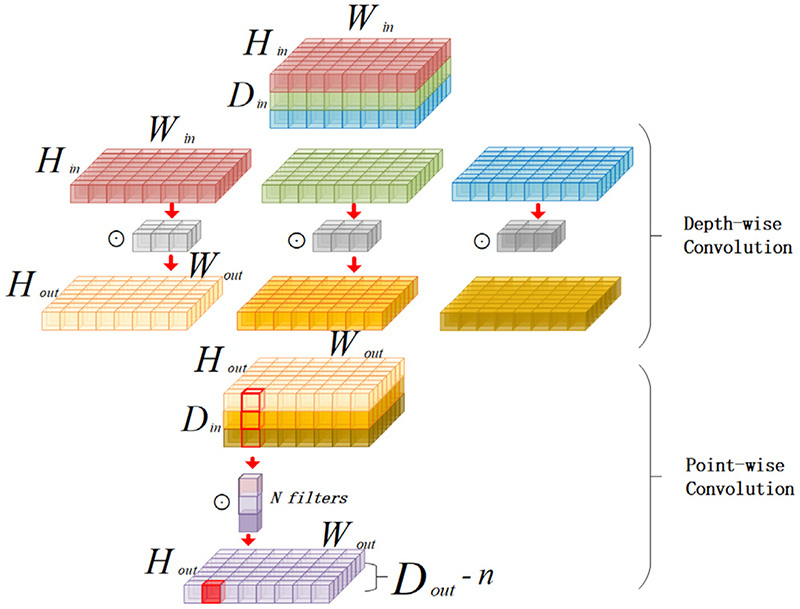
Structure of depth-wise and point-wise convolution.

**Fig. 7:**
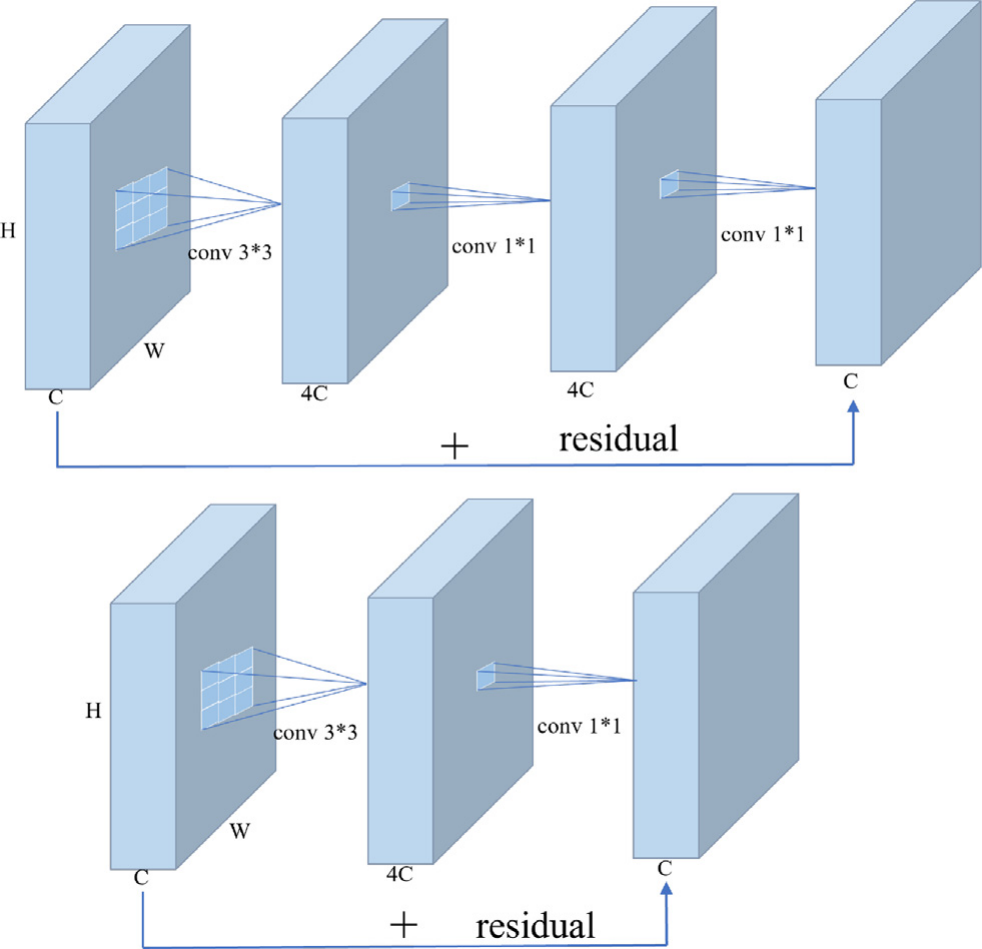
Fused inverted residual.

Depth-wise separable convolution used above is a technique for reducing parameters, which corresponds to the depth-wise convolution and point-wise Convolution in Fig. 5. The specific structure is shown in Fig. 6. The depth-wise separable convolution splits an ordinary 3 x 3 convolution into two convolutions. The first convolution applies a 3 x 3 convolution to each input channel. A convolution kernel convolves one channel. This operation is called depth-wise convolution. Another convolution applies a 1 x 1 kernel to all channels to generate a new feature map by weighting the combination of previous feature maps in the depth direction, which is called point-wise convolution. Depth-wise separable convolution is the same as ordinary 3 x 3 convolution transformation, with the advantage of reducing the parameters. However, its operating efficiency still needs to be improved. Therefore, we propose the Fused Inverted Residual, as shown in Fig. 7. We fuse the first 3 x 3 convolution and the second 1 x 1 convolution of the upper inverted residual block into one 3 x 3 convolution to get the lower side of the Fused Inverted Residual, which solves the slow problem caused by the depth-wise convolution. Meanwhile, it can accelerate the operation of the PA convolution block.

**Fig. 8:**
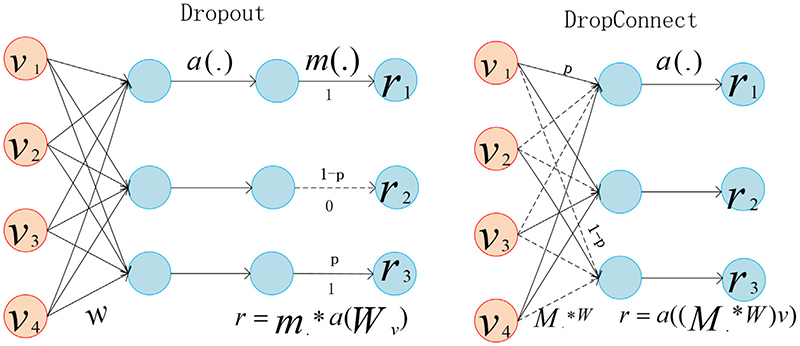
Dropconnect Block.

To introduce weight sparsity, we propose DropConnect operation in PA convolution block instead of Dropout, reduce overfitting and improve performance. As shown in Fig. 8, the output of hidden layer nodes is not randomly cleared to 0 but instead clears the input weight of each node connected to it with 1 – p probability. In Fig. 8, v is the input layer, and r is the output layer, both of which are n x 1 dimensional column vectors. W is a multi-dimensional matrix of weight parameters, a(x) is the form of excitation function satisfying a(0) = 0, m is a column vector composed of 0, 1, and the multiplication of m and a(W_v_) is the multiplication of corresponding elements. The right side is similar, where M is the binary matrix used to encode the connection information.

### 3.4 Relative self-attention transformer block

Transformer (Vaswani et al., 2017) is a self-attention mechanism for learning the relationship between sequence elements. The Relative self-attention transformer block based on the attention head is proposed to utilize the relative positions and distances between sequence elements effectively. It can form the output of the self-attention sublayer. Self-attention sublayer adopts *h* attention heads. Each head concatenated and parametric linear transformation is applied to obtain the output of the sublayer:

(11) $${\text{z}}_{\text{i}}=\sum_{\text{j}=1}^{\text{n}}\alpha_{\text{ij}}({\text{x}}_{\text{j}}{\text{W}}^{\text{V}}).$$

In Eq. (11), each attention head operated on n elements of input patch x = (x1, ‥, x_n_), where x_i_ ∈ R^d_x_^, and computed new sequence z = (z_1_,‥, z_n_), where z_i_ ∈ R^d_z_^.

To propagate relative information of input patch to sublayer outputs, we modify Eq. (11):

(12) $${\text{Z}}_{\text{i}}=\sum_{\text{j}=1}^{\text{n}}\alpha_{\text{ij}}({\text{x}}_{\text{j}}{\text{W}}^{\text{V}}+\alpha_{\text{ij}}{\;}^{\text{V}}),$$

where α_ij_ is weight coefficient, calculated by Eq. (13) using the soft-max function:

(13) $$\alpha_{\text{ij}}=\frac{\text{exp}({\text{e}}_{\text{ij}})}{\sum_{\text{k}=1}^{\text{n}}\text{exp}({\text{e}}_{\text{ik}})},$$

(14) $${\text{e}}_{\text{ij}}=\frac{{\text{x}}_{\text{i}}{\text{W}}^{\text{Q}}{\left({\text{x}}_{\text{j}}{\text{W}}^{\text{K}}+{\text{a}}_{\text{ij}}^{\text{K}}\right)}^{\text{T}}}{\sqrt{{\text{d}}_{\text{z}}}}.$$

**Fig. 9:**
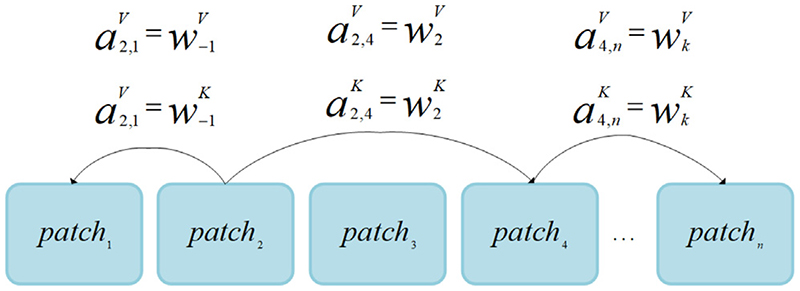
Relative positions with example patches.

In Eq. (14), the relative information between the input patch x_i_ and x_j_ is represented by vector ${\text{a}}_{\text{ij}}^{\text{V}},{\text{a}}_{\text{ij}}^{\text{K}}\in {\text{R}}^{\text{da}}$. e_ij_ is calculated by comparing the compatibility function of the two input elements, where W^Q^, W^K^, W^V^ are parameter matrices that used for each layer and attention head. In the relative self-attention transformer, the relative position between patches replaces the absolute position. Fig. 9 shows some example patches with relative positions and distances between elements. We learn a representation for the relative position within k distance.

When considering relative positions, the representations of different position pairs are different. This makes it impossible to compute all e_ij_ for all positions with single matrix multiplication. Therefore, we decompose Eq. (14) into two items to solve:

(15) $${\text{e}}_{\text{ij}}=\frac{{\text{x}}_{\text{i}}{\text{W}}^{\text{Q}}{\left({\text{x}}_{\text{j}}{\text{W}}^{\text{K}}\right)}^{\text{T}}+{\text{x}}_{\text{i}}{\text{W}}^{\text{Q}}{\left({\text{a}}_{\text{ij}}^{\text{K}}\right)}^{\text{T}}}{\sqrt{{\text{d}}_{\text{z}}}}.$$

In Eq. (15), the first term is the same as Eq. (14) and can be calculated as above. The second term is the representation of relative position, which can use tensor reshaping to compute n parallel multiplications of the matrix. Each matrix multiplication computes all head and batch contributions to e_ij_, corresponding to a particular sequence position. The relative self-attention transformer block introduces the relative position information in the calculation process, thus breaking the Permutation-invariant property of selfattention (Vaswani et al., 2017) and improving the relationship construction between patches.

### 3.5 Intermittent fully connected layer

The size of the FC layer's hidden layer is critical. A largely hidden layer with more parameters usually improves the prediction accuracy but dramatically increases the number of weights. And a small hidden layer does not propagate all input features well, resulting in suboptimal results. To make up for both shortcomings and solve the parameter redundancy and overfitting caused by a fully connected layer, we propose an IFC layer.

**Fig. 10:**
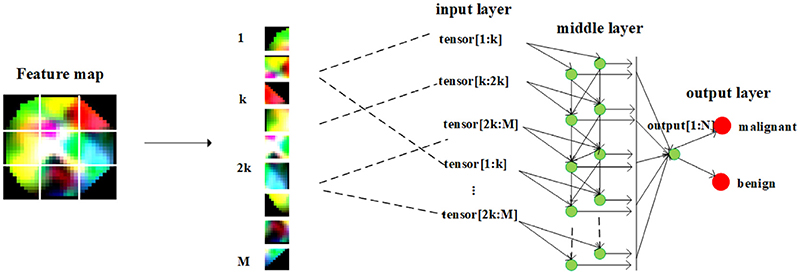
IFC layer.

The architecture of IFC is shown in Fig. 10, which includes the input layer, middle layer, and output layer. First, the feature map obtained from the transformer block is split into 1-k, k-2 k, 2 k-M, where k is the hyperparameter needed to set, and M is the size of the entire input. We adopted the step-by-step repeating input mode, consisting of multiple split data for the input layer. The middle layer of IFC consists of different hidden layers, each composed of multiple neurons. The output layer is also composed of multiple neurons based on classification numbers. The number of interneurons is usually kept small to reduce the number of multiplications. Since the number of middle hidden neurons is usually small, the network may underfit. Therefore, we make each layer repeatedly receive input from the previous layer to preserve certain features of the middle layer. The progressive and repeated input functions enabled the neural network to achieve the desired results with fewer parameters, improving performance with faster responses.

### 3.6 Gradient awareness minimization

To solve the scale-dependent problem and improve the generalization performance, we introduce the concept of scale-invariant adaptive sharpness and propose a novel learning method named GAM.

In GAM, gradient-aware sharpness is adopted to minimize the corresponding generalization bound, which could avoid the scale-dependent problem faced by SAM (Foret et al, 2010). Based on the relation between the generalization metric and loss minimization, we propose an adaptive sharpness of the loss function and define the max sharpness region determined by the normalization operator. Specifically, we use scale-invariant gradient-aware sharpness measures to overcome the side-effect of scale dependence caused by sharpness training. Similarly, the generalization bound can be obtained via gradient-aware sharpness derived from the Eq. (16) of SAM (Foret et al, 2010).

(16) $${\text{L}}_{\text{D}}(\text{W})\leq \underset{\|\varepsilon {\|}_{\text{p}}\leq \text{r}}{\text{max}}{\text{L}}_{S}(\;\text{W}+\varepsilon )+\text{h}(\frac{\|\text{W}{\|}_{2}^{2}}{{\text{r}}^{2}}),$$

(17) $${\text{L}}_{\text{D}}(\text{w})\leq \underset{\|{\text{N}}_{\text{w}}^{-1}\varepsilon {\|}_{2}\leq \text{r}}{\text{max}}{\text{L}}_{\text{S}}(\text{w}+\varepsilon )+\text{h}(\frac{\|\text{w}{\|}_{2}^{2}}{{\text{n}}^{2}{\text{r}}^{2}}).$$

In Eq. (17), ${\text{N}}_{\text{w}}^{-1}$ is the normalization operator of R^k^, h is a strictly increasing function on R^k^ → R^k^, $\text{n}=|\text{S}|,\text{r}=\sqrt{\text{k}}\sigma (1+\sqrt{\text{log}\;\text{n}/\text{k}})$. The generalization bound in the right side of Eq. (17) can be described by gradient-aware sharpness:

(18) $$(\underset{\|{\mathbb{N}}_{\text{w}}^{-1}\varepsilon {\|}_{\text{p}}\leq \text{r}}{\text{max}}{\text{L}}_{\text{S}}(\text{w}+\varepsilon )-{\text{L}}_{\text{S}}(\text{w}))+\text{h}(\frac{\|\text{w}{\|}_{2}^{2}}{{\text{n}}^{2}{\text{r}}^{2}}).$$

Since $\text{h}(\frac{\|\text{w}{\|}_{2}^{2}}{{\text{n}}^{2}{\text{r}}^{2}})$ is a strictly increasing function with $\|\text{w}{\|}_{2}^{2}$, it can be viewed as a standard L_2_ regularization term (Foret et al, 2010). Therefore, the gradient awareness minimization problem can be defined as:

(19) $$\underset{\text{w}}{\text{min}}\;\underset{\|{\text{N}}_{\text{w}}^{-1}\varepsilon {\|}_{\text{p}}\leq \text{r}}{\text{max}}{\text{L}}_{\text{S}}(\text{w}+\varepsilon )+\frac{\lambda }{2}\|\text{w}{\|}_{2}^{2}.$$

To solve the minimax problem in Eq. (19), we find the optimum parameter ε. Similar to SAM (Foret et al, 2010); ε can be approximated to maximize L_S_(ω + ε) by first-order approximation method.

Since $\underset{\|\tilde{\varepsilon }{\|}_{\text{p}}\leq \text{r}}{\text{argmax}}\;{\text{L}}_{\text{s}}({\text{W}}_{\text{t}}+{\text{N}}_{{\text{w}}_{\text{t}}}\tilde{\varepsilon })\approx \underset{\|\tilde{\varepsilon }{\|}_{{\|}_{\text{p}}}\leq \text{r}}{\text{argmax}}\;{\tilde{\varepsilon }}^{\text{T}}{\text{N}}_{{\text{w}}_{\text{t}}}\nabla {\text{L}}_{\text{s}}({\text{W}}_{\text{t}})$, the following formula can be obtained: Table 1

(20) $$\begin{array}{l} {\tilde{\varepsilon }}_{\text{t}}=\underset{\|\varepsilon ∼{\|}_{\text{p}}\leq \text{r}}{\text{argmax}}\;{\text{L}}_{S}({\text{w}}_{\text{t}}+{\text{N}}_{{\text{w}}_{\text{t}}}\tilde{\varepsilon })\\ \;=\text{r}\times \text{sign}(\nabla {\text{L}}_{\text{s}}({\text{W}}_{\text{t}}))\frac{|{\text{N}}_{{\text{w}}_{\text{t}}}\nabla {\text{L}}_{\text{s}}({\text{W}}_{\text{t}}){|}^{\text{q}-1}}{{\|\text{N}}_{{\text{w}}_{\text{t}}}\nabla {\text{L}}_{\text{s}}({\text{W}}_{\text{t}}{\|}_{\text{q}}^{\text{q}-1}}, \end{array}$$

where $\tilde{\varepsilon }={\text{N}}_{\text{W}}^{-1}\varepsilon$. Then, the two-step process of GAM can be described as:

(21) $$\left\{ \begin{array}{c} \varepsilon_{\text{t}}=r\times {\text{N}}_{{\text{w}}_{\text{t}}}\;\text{sign}(\nabla {\text{L}}_{\text{S}}({\text{W}}_{\text{t}}))\frac{|{\text{N}}_{{\text{w}}_{\text{t}}}\nabla {\text{L}}_{\text{s}}({\text{W}}_{\text{t}}){|}^{\text{q}-1}}{\|{\text{N}}_{{\text{w}}_{\text{t}}}\nabla {\text{L}}_{\text{s}}({\text{W}}_{\text{t}}){\|}_{\text{q}}^{\text{q}-1}}\\ \;{\text{W}}_{\text{t}+1}={\text{W}}_{\text{t}}-\alpha_{\text{t}}(\nabla {\text{L}}_{\text{S}}({\text{W}}_{\text{t}}+\varepsilon_{\text{t}})+\lambda {\text{W}}_{\text{t}}) \end{array}.\right.$$

Table 2 shows the principle and process of the GAM algorithm. The algorithm's input is the MRI image of the training set, and the algorithm's output is the trained weight of the model. First, we set the hyperparameter p to 2. For the input, we first define the loss function and then set the radius of maximization region r, weight decay coefficient λ, and learning rate α. GAM solves the minimax problem by iteratively applying a two-step procedure for *t* = 0, 1, 2, … by Eq. (21). For *t* = 0, 1, 2, …, *n*, especially if p = 2, the calculation formula of ε can be obtained as illustrated in Table 2. In the rigid region with fixed radius r, GAM estimates the point w_t_ + ε_t_ at which the loss is approximately maximized and perform gradient ascent around w_t_, then performs gradient descent at w_t_ using the gradient at the maximum point w_t_ + ε_t_. In the training phase, the optimal ε is calculated iteratively for each batch, and the initialized weight w is updated according to ε until the algorithm get convergence.

## 4 Experiment

### 4.1 Experimental settings

Our experiment is performed on NVIDIA QUADRO RTX 8000, whose CUDA version is 10.2. GPU memory of the server is 48 GB, and the memory type is GDDR6. All experiments were based on python 3.9. The framework adopted in the experiment is Pytorch (Paszke, et al., 2019) and scikit-learn (Pedregosa et al, 2011).

### 4.2 Performance measures

**Fig. 11:**
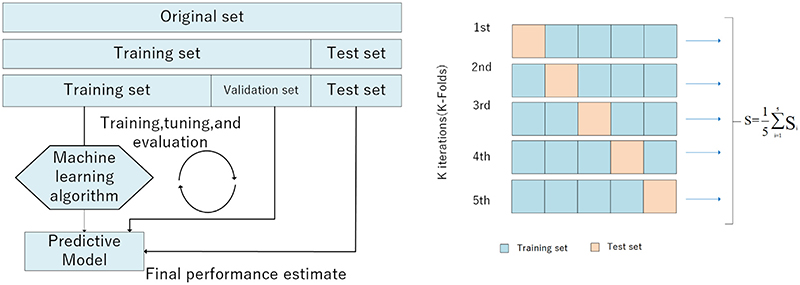
K-fold process.

K-fold cross-validation is commonly employed to test the accuracy of the classification model. We divide the BraTS 2019 dataset into five parts and use four parts as a train set and one as test data in each round of algorithm experiments, which is shown in Fig. 11. The advantage of this operation is that ratio could be maintained at 8:2, which is better for guaranteeing the size of the test set. Each trial yields a correct rate. It treats an average of 5 correct rates as an estimate of accuracy.

Five evaluation metrics have been employed to evaluate our method: precision, recall, specificity, F1-score, and accuracy. They are defined as follows:

(22) $$\;\text{Precision}\;=\frac{\text{TP}}{\text{TP}+\text{FP}}$$

(23) $$\;\text{Recall}\;=\frac{\text{TP}}{\text{TP}+\text{FN}}$$

(24) $$\;\text{Specificity}\;=\frac{\text{TN}}{\text{FP}+\text{TN}}$$

(25) $$\text{F}1=\frac{2\times \;\text{Precision}\;\times \;\text{Recall}\;}{\;\text{Precision}\;+\;\text{Recall}\;}$$

(26) $$\text{Accuracy}\;=\frac{\text{TP}+\text{TN}}{\text{TP}+\text{FN}+\text{TN}+\text{FP}}$$

### 4.3 Settings of hyperparameters

The setting of hyperparameters is particularly important, and it is generally determined by experience, such as batch size and learning rate.

Batch Size is the number of samples selected for a training session. The Batch Size affects the degree and speed of model optimization and directly affects the GPU memory usage. GPU to the power of 2 of the batch can play a better performance, so set to 16, 32, 64,128…, which is often better than when set as a multiple of 10 or 100. In our study, when setting Batch Size, a larger Batch Size is first selected to fill up the GPU, and the loss convergence is observed. If there is no convergence or the convergence effect is not good, the Batch Size will be reduced. Finally, we select a Batch Size of 64.

The initial learning rate plays a decisive role in the convergence of the deep network. If the initial learning rate is too low, the loss of network will decline very slowly; if the initial learning rate is too large, the range of parameter updating will be very large, which will lead to the convergence to the local-optimal solution, or the loss will directly start to increase.

The selection strategy of the learning rate is constantly changing in the process of network training. In the beginning, the parameters are relatively random, so we should choose a relatively large learning rate, so that loss will decrease faster. After training for a period of time, the update of parameters should have a smaller range, so the learning rate generally attenuates.

There are many ways of attenuating, the way we adopt one of the exponential attenuation methods, StepLR and its specific steps are shown in Table 3. First, we initialize the learning rate to α_0_ = 0.01, then adjust the current learning rate α according to the current epoch n and the number of total epochs N for training.

### 4.4 Exploration of the best combination by optimization algorithm

We first investigate the influence of the stack way of convolution and attention blocks for optimal performance. Convolutions perform down-sampling and global relative attention operations only when the feature maps are small enough to be processed. There are two ways to do down-sampling. The first is to divide the image into blocks, as in the ViT model (Dosovitskiy et al,., 2010), and stack-related self-attention blocks. The second is a multi-stage operation with progressive pooling.

**Fig. 12:**
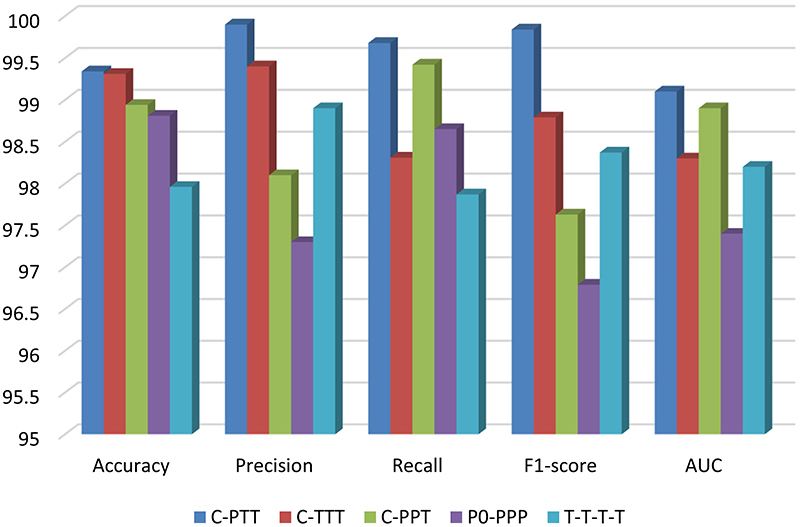
Corresponding indicators for different combinations.

Our methods can be described in four stages. The first stage, called C, consists of classical convolutions and PA convolution blocks to achieve dimensionality reduction. The last three stages consist of convolutional blocks or Transformer blocks, resulting in 5 combinations: CPTT, CTTT, CPPT, CPPP, and TTTT, where P represents the PA convolution block, and T represents the transformer block. Table 4 shows the detailed metrics obtained for different combinations, and the schematic diagram of each indicator is illustrated in Fig. 12. The accuracy, precision, recall, and F1-score values of CPTT are 99.34 %, 99.9 %, 99.68 %, and 99.84 %, respectively. Compared with other combinations of CTTT, CPPT, CPPP, and TTTT, CPTT has the best performance in all indicators.

### 4.5 Exploration of the best numbers of blocks and channels by optimization analysis

In this section, we analyze the impact of GAM on SpCaNet further by permuting and combining different numbers of blocks and channels. As shown in Table 5, N represents the number of each module. These modules include classical convolution, PA convolution, and transformer. C represents the number of feature map channels passed in by each module. Block vs channels 1−5 represent different combinations of classical convolution, PA convolution, and transformer. For the number of channels L1 to L4, we use incremental doubling while ensuring that Stem L0 has a smaller or equal width to be the same as L1. For simplicity, only the number of blocks in L2 and L3 are scaled when increasing the network depth.

**Fig. 13:**
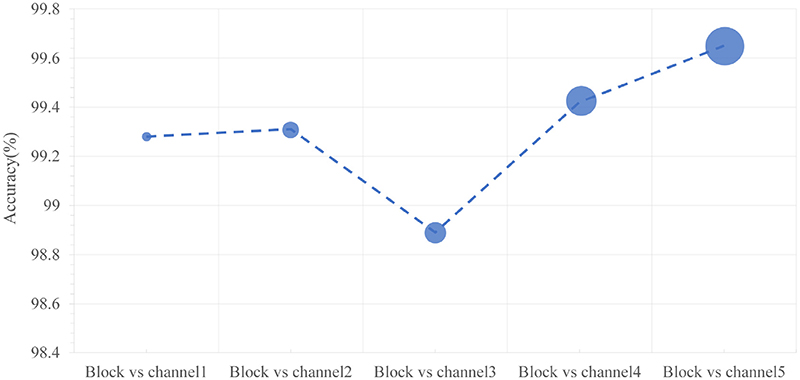
Accuracies with different numbers of blocks and channels.

In Fig. 13, the size of the dots markers represents the number of parameters, and the accuracy increases slightly from Block vs channel 1 to Block vs channel5. The accuracy of Block vs channel1 is 99.28 %, which is similar to the performance of other schemes with 18.2 M parameters. The parameters of other combinations are 33.6 M, 56.1 M, 118 M, and 205 M, respectively, which are nearly twice as likely to Block vs channel1. Considering the balance of accuracy and number of parameters, we use ‘Block vs channel1’ in the following experiments.

### 4.6 Evaluation of RA and IFC with individual and hybrid strategies

To demonstrate the performance of the proposed RA and IFC, we performed a series of ablation experiments, the corresponding results of which are listed in Table 6. These experiments show that in the case of comparable computational cost, positional information embedding is more conducive to classifying brain tumor images, and RA achieves the greatest improvement in accuracy by 2.0 %. RA inherits the advantages of extrusion and excitation attention from channel attention methods that simulate relationships between channels while capturing long-distance dependencies with precise positional information. Experiments in the classification of brain tumors demonstrated the effectiveness of RA. In the comparison experiment between “+RA + IFC” and other experiments, it can be seen that the performance of the proposed IFC further improves the performance. Its parameter amount is reduced to 18.21 M from 25.97 M compared with the FC in the baseline. Experiments show that IFC achieves higher classification accuracy of brain tumor images while reducing computational costs.

### 4.7 Evaluation of SpCaNet

To verify the effectiveness of the proposed SpCaNet, we implement comparative experiments on BraTS2019 with single convolutional networks, Vit-stem Transformer networks, Multi-stage Transformer networks, and Conv + TFM networks, including effi-cientnetv2_l (Tan and Le, 2019); ResNet152 (He, et al., 2016); DeiT (Touvron et al, 2021); ViT-B/16 (Dosovitskiy et al, 2010); ViT-L/16 (Dosovitskiy et al, 2010); Swin-B (Liu, et al., 2021); Swin-L (Liu, et al., 2021); BotNet (Srinivas, et al., 2021).

Each method’s results are shown in Table 7, where FLOPs represent the number of floating-point operations, and params represent the size of the model parameters. By observing each model’s accuracy and other indicators, it could be found that SpCaNet achieves the best performance. The design of the PA convolution block and IFC makes SpCaNet more lightweight. Compared with the FLOPs of other models ranging from 11443.2 to 59669.8 M, the FLOPs of SpCaNet-1 and SpCaNet-2 are 3336.8 M and 6846.4 M, respectively, which significantly reduces floating-point operations.

**Fig. 14:**
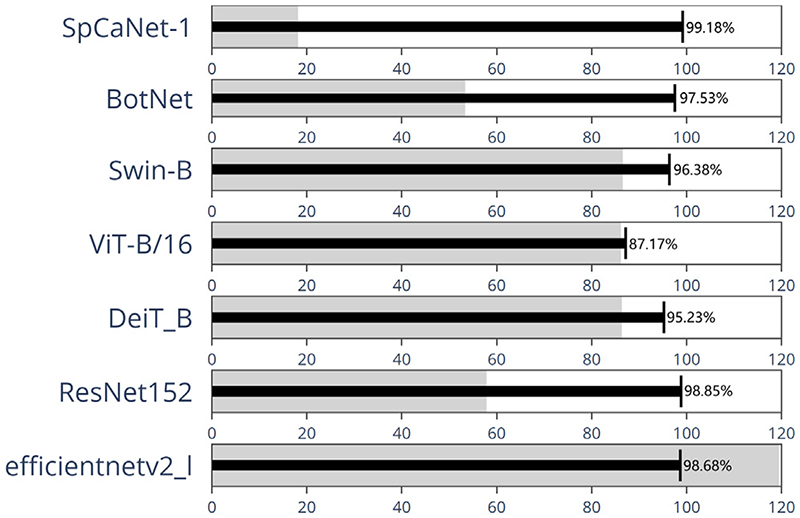
The comparison between our model with each model.

As is shown in Fig. 14, we use a bullet chart to briefly show the accuracy and parameters of each model, in which the thin black line represents the accuracy rate, and the thick gray line represents the number of parameters. For example, the accuracy rate of SpCaNet-1 is 99.18 %, and the number of its parameters is 18.2 M. Compared with BotNet (53.4 M), the parameter amount of SpCaNet-1 is reduced by more than three times. Compared with ViT-L/16 (326.4 M), the number of SpCaNet-1 parameters is reduced by about eighteen times. From the comparison of each model, it can be inferred that SpCaNet has achieved the best performance in feature extraction.

### 4.8 Evaluation of GAM-SpCaNet

To verify the effectiveness of the proposed GAM, we compare it with the SGD optimizer. The accuracy rates and the difference between the accuracy rates of the test and training sets are shown in Table 8. For example, DeiT_B has an accuracy of 95.23 % at 50 epochs under SGD, and the difference between the training set and test set is 2.6 ± 0.47 %. In the comparison test with SGD, the superior performance of GAM has a significant performance benefit. In general, the accuracy of each model is improved by 1 % at 200 epochs, such as DeiT_B, ViT-B/16, ViT-L/16, and BotNet. Furthermore, with increasing training epochs, GAM can continue improving accuracy without overfitting.

**Fig. 15:**
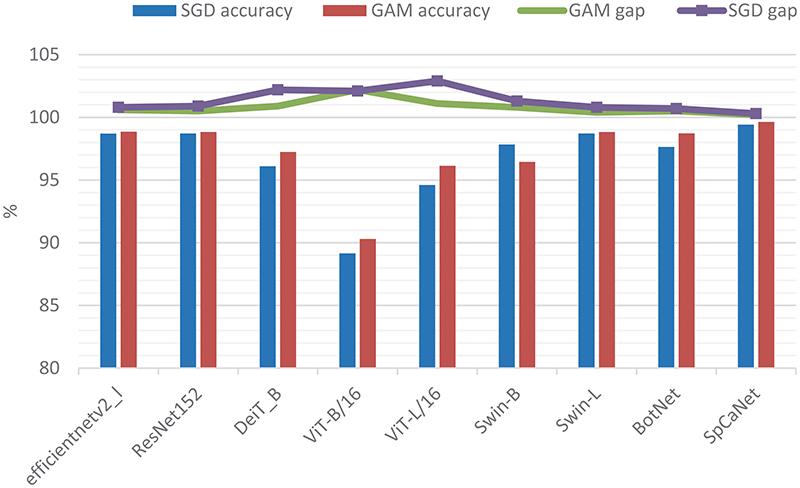
Comparison of the accuracy and gap between the test set and training set.

On the contrary, the standard training method without GAM often overfits when training multiple epochs. In Fig. 15, the curve is the difference value between the test set and the training set, and the bar graph represents the accuracy value. GAM dramatically reduces the accuracy difference between the training and test sets. It can also reduce the risk of overfitting to a certain extent, especially for DeiT_B and Vit_L/16.GAM achieves excellent results with its scale-invariant gradient-aware sharpness, which improves the training path in the weight space by adjusting the maximized region relative to the weight scale. The experimental results confirm that GAM helps to improve model performance and generalization ability.

In the evaluation of GAM-SpCaNet, 5-fold cross-validation is performed, and the results are as follows in Table 9, which describes the model's specificity, precision, recall, and F1-Score values. The performance measures for malignant were 100 %, 100 %, 99.66 %, and 99.83 %, respectively. According to Table 10, the overall average accuracy of the system is 99.28 ± 0.34 %.

**Fig. 16:**
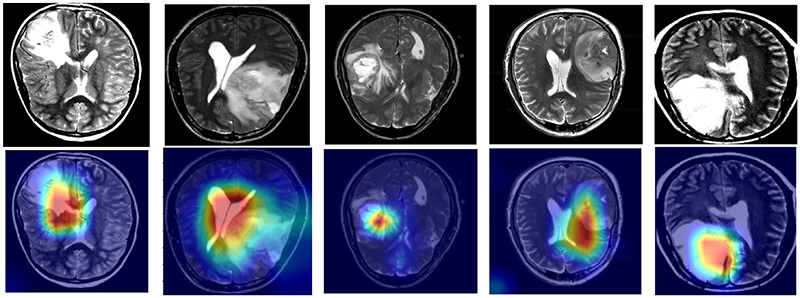
The visualization of our model with Grad-CAM.

**Fig. 17:**
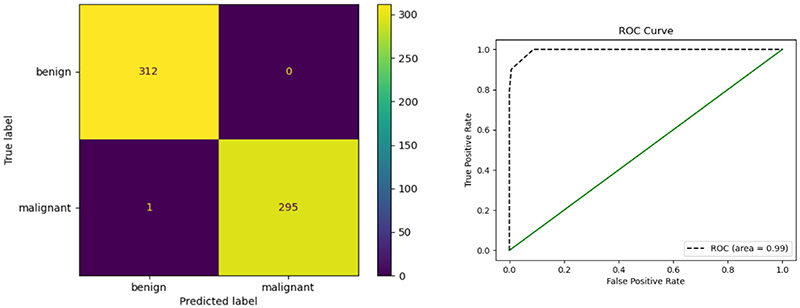
Confusion matrix and ROC Curve.

Fig. 16 shows the visualization of GAM-SpCaNet on Grad-CAM, the parameters of the last layer Transformer of SpCaNet are selected to combine with Grad-CAM. It can be found that GAM-SpCaNet can accurately focus on the lesion area. The confusion matrix plot and ROC curve are shown in Fig. 17. It could be seen our model has excellent performance.

### 4.9 Comparison with the state-of-art methods

To verify the effectiveness of GAM-SpCaNet, we conducted the comparative experiment with four SOTA methods (SE-ResNeXt-MLT (Linqi and Jingyang, 2022), FCNN-CRFs (Gull and Khan, 2021), KSVM-SSD (Rao, 2022), Ensemble (Breiman, 2017). These results were obtained from the corresponding papers, which were implemented on the same dataset of BraTS2019. The comparative results are shown in Table 11. Compared with SOTA models (SE-ResNeXt-MLT: 98.99 %; FCNN-CRFs: 97.31 %; KSVM-SSD: 98.84 %; Ensemble: 97 %), GAM-SpCaNet has the highest accuracy (99.28 %). It can also be inferred that GAM-SpCaNet has excellent advantages in other indicators (Specificity:100 %, Recall:99.66 %, Precision:100 %). From the experiment results, it can be found that our method has an excellent overall discriminative ability and performs well in all indicators, which indicates that our method has stronger diagnostic ability for patients with suspected brain tumors.

### 4.10 Statistical test analysis

Table 12 and Table 13 have shown the results of the independent sample *t*-test, including the results of mean ± standard deviation, t-test results, significance P value, and Cohen's d value for effect size. For Table 12, we randomly sample 16 samples of the SOTA models as well as 16 samples of the SpCaNets. For Table 13, we randomly sample 45 samples under SGD and samples under GAM, respectively.

The mean values of SOTA models and SpCaNets on Accuracy are 98.358/99.069; The P value of the F test is 0.003 < 0.05, so the statistical results are significant, indicating that SOTA models and SpCaNets have significant differences in Accuracy. Cohen's d value I of the difference is 1.126, which is very large (0.20, 0.50, and 0.80 correspond to small, medium, and large critical points, respectively). The mean values of Precision of SOTA models and SpCaNets are 97.65/98.314; The P value of the F test is 0.046 < 0.05, so the statistical result is significant, indicating that there is a significant difference in Precision between SOTA models and SpCaNets. Cohen's d value of the difference was 0.735, and the difference was medium. It can be seen from Table 12 that SpCaNets and SOTA models have obvious differences in the two indicators, which represents the superiority of SpCaNets.

The mean values of SGD and GAM on Accuracy under 50 epochs are 95.696/97.129, respectively. The P value of the F test is 0.048 < 0.05, indicating that there is a significant difference between SGD and GAM under 50 epochs. The mean values of SGD and GAM on Accuracy under 200 epochs are 95.941/97.282, respectively. The P value of the F test is 0.037 < 0.05, so the statistical result is significant under 200 epochs. Cohen's d value of the difference is 0.446, which is near medium. It can be obtained from the significant difference that GAM has a certain improvement over SGD to a certain extent.

## 5 Conclusion

Accurate brain tumor detection is still challenging because of the irregular shape and variable appearance of brain tumors. Existing work has limitations for identifying the substructure of tumor regions and classifying malignant and benign images. GAM and SpCaNet are proposed to diagnose the brain tumor. Experimental results confirm that our method is superior to eight SOTA CNN and Transformer models and exceeds four SOTA brain-tumor diagnosis models.

Our GAM-SpCaNet has the best performance because (i) SpCa-Net leverages the power of both convolutional neural network (CNN) and transformer. Based on the PA convolution block's translation equivariance and the global receptive field of relative self-attention, SpCaNet can better extract pathological features. (ii) The proposed GAM optimizes the model training process, which significantly increases the generalization characteristics of the model. (iii) The lightweight of the PA convolution block and IFC helps reduce training time and improve training efficiency.

In future work, we will further optimize the SpCaNet variant to reduce the hardware resource requirements. Meanwhile, we will optimize the interpretability of the model better to show the detailed process of brain tumor recognition.

## Figures and Tables

**Table 1:** Recent algorithms for Brain tumor diagnosis and detection.

Author	Model	Dataset	Aim of study
Zhou et al. (Zhou, 2018)	DenseNet-LSTM	422 MRI scans containing normal control images as well as three types of brain tumors	Diagnosis and prognosis
Chang et al. (G. J. Chang P, Weinberg B D,, 2018)	2D/3D hybrid CNN	MR imaging data and molecular data of 259 patients with gliomas	Diagnosis
Yang et al. (Y. L. F. Yang Y, Zhang X,, 2018)	AlexNet and GoogLeNet with pre-trained CNN	52 patients (grade II: 25, grade III: 27) with LGG and 61 patients with HGG	Detection
Jiang et al. (C. N. Linqi J, Jingyang L,, 2022)	SE-ResNeXt	BraTS2019 dataset	Detection
Gull et al. (A. S. Gull S, Khan H U,, 2021)	fully convolutional neural network (FCNN) and transfer learning techniques	BraTS2019 dataset	Detection
Rao et al. (K. K. Rao C S,, 2022)	kernel support vector machine (KSVM) and social ski driver (SSD) algorithm	BraTS2019 dataset	Diagnosis

**Table 2:** Schematic diagram of the GAM algorithm.

Algorithm: Gradient Awareness Minimization (*p* = 2)
**Input:** Training dataset $\text{S}≔{\text{U}}_{\text{i}=1}^{\text{n}}\{({\text{x}}_{\text{i}},{\text{y}}_{\text{i}})\}$,
loss function l, batch size b,
the radius of maximization region r,
weight decay, coefficient λ,
scheduled learning rate α, initial weight w_0_.
**Output:** A model with trained weight w.
**Initialize weight:** w ≔ w_0_,
**While** the model does not converge, **do**
Sample a mini-batch B of size b from S,
$\varepsilon ≔\text{r}\frac{{\text{N}}_{\text{w}}^{2}\nabla {\text{L}}_{\text{B}}(\text{w})}{\|{\text{N}}_{\text{w}}\nabla {\text{L}}_{\text{B}}(\text{w}){\|}_{2}},$
$\text{W}≔\text{W}-\alpha (\nabla {\text{L}}_{\text{B}}(\text{W}+\varepsilon )+\lambda \text{W}).$
**end while**
**return** w

**Table 3:** The pseudocode of the settings of the learning rate of StepLR.

The procedure of StepLR
**Input:**
The number of total epochs N,
initial learning rate α_0_,
current epoch n.
**Output:** current learning rate α.
**if** $\text{n}<\text{N}\times \frac{3}{10}$, **do**:
$\alpha ≔\alpha_{0}\text{elif}\;\text{n}<\text{N}\times \frac{6}{10}$, **do**:
$\alpha ≔\alpha_{0}\times 0.2\text{elif}\;\text{n}<\text{N}\times \frac{8}{10}$, **do:**
α ≔ α_0_ × 0.2^2^**else, do:**
α ≔ α_0_ × 0.2^3^
**endif**
**return** α.

**Table 4:** Comparison of different Transformer and PA combinations.

Combination	Accuracy (%)	Precision (%)	Recall (%)	F1-score (%)	AUC
CPTT	99.34 ± 0.31	99.94	99.68	99.81	0.991
CTTT	99.31 ± 0.53	99.40	98.31	98.85	0.983
CPPT	98.94 ± 0.29	98.08	99.42	98.75	0.989
CPPP	98.81 ± 0.76	97.32	98.65	97.98	0.974
TTTT	97.96 ± 0.73	98.90	97.87	98.38	0.982

**Table 5:** Different combinations of blocks and channels.

Setting	L0-Conv	L1-PAConv	L2-PAConv	L3-TF	L4-TF	Accuracy	Param. (M)
Block vs channel1	N = 2,C = 64	N = 2,C = 96	N = 3,C = 192	N = 5,C = 384	N = 2,C = 768	99.28 ± 0.38	18.2
Block vs channel2	N = 2,C = 64	N = 2,C = 96	N = 6,C = 192	N = 14,C = 384	N = 2,C = 768	99.31 ± 0.65	33.6
Block vs channel3	N = 2,C = 128	N = 2,C = 128	N = 6,C = 256	N = 14,C = 512	N = 2,C = 1024	98.89 ± 0.67	56.1
Block vs channel4	N = 2,C = 192	N = 2,C = 192	N = 6,C = 384	N = 14,C = 768	N = 2,C = 1536	99.42 ± 0.34	118
Block vs channel5	N = 2,C = 192	N = 2,C = 192	N = 12,C = 384	N = 28,C = 768	N = 2,C = 1536	99.65 ± 0.26	205

(N represents the number of each module, C represents the number of feature map channels passed in by each module).

**Table 6:** Classification results under different methods with the SpCaNet baseline.

Setting	Param. (M)	Accuracy (%)
Baseline	25.68	96.5
+SE	25.87	97.6_+1.1_
+CBAM	25.88	97.8_+1.3_
+RA	25.97	98.5_+2.0_
+RA + IFC	18.21	99.1_+2.6_

(A_+B_ means the accuracy is A which improves the baseline accuracy by B).

**Table 7:** The Comparison with other convolution and transformer models.

Setting	Model	Eval Size	Param. (M)	FLOPs (M)	Accuracy (%)	Precision (%)	Recall (%)	f1score (%)	AUC	CM
Conv only	efficientnetv2_l (Tan and Le, 2019)	224^2^	119.5	12333.8	98.68	94.55	99.66	97.04	0.98	$\left[\begin{array}{cc} 305 & 7\\ 1 & 295 \end{array}\right]$
	ResNet152 (He, et al., 2016)	224^2^	57.9	11539.5	98.85	100	97.64	98.81	0.99	$\left[\begin{array}{cc} 312 & 0\\ 7 & 289 \end{array}\right]$
ViT-Stem TFM	DeiT_B (H. Touvron, et al, “Training data-efficient image transformers distillation through attention,” International Conference on Machine Learning. PMLR, 2021)	224^2^	86.4	16848.7	95.23	97.17	92.91	94.99	0.98	$\left[\begin{array}{cc} 304 & 8\\ 21 & 275 \end{array}\right]$
	ViT-B/16 (B. L. Dosovitskiy A, Kolesnikov A, et al, “An image is worth 16x16 words: Transformers for image recognition at scale[J],” arXiv preprint arXiv:, et al., 2010)	224^2^	86.2	16848.4	87.17	91.29	81.42	86.07	0.87	$\left[\begin{array}{cc} 289 & 23\\ 55 & 241 \end{array}\right]$
	ViT-L/16 (B. L. Dosovitskiy A, Kolesnikov A, et al, “An image is worth 16x16 words: Transformers for image recognition at scale[J],” arXiv preprint arXiv:, et al., 2010)	224^2^	326.4	59669.8	96.22	97.23	94.93	96.07	0.95	$\left[\begin{array}{cc} 304 & 8\\ 15 & 281 \end{array}\right]$
Multi-stage TFM	Swin-B (Liu, et al., 2021)	224^2^	86.6	15125.5	96.38	93.91	98.99	96.38	0.96	$\left[\begin{array}{cc} 293 & 19\\ 3 & 293 \end{array}\right]$
	Swin-L (Liu, et al., 2021)	224^2^	194.8	34017.6	99.18	99.66	98.65	99.15	0.99	$\left[\begin{array}{cc} 311 & 1\\ 4 & 292 \end{array}\right]$
Conv + TFM	BotNet (Srinivas, et al., 2021)	224^2^	53.4	11443.2	97.53	96.99	97.97	97.48	0.97	$\left[\begin{array}{cc} 303 & 9\\ 6 & 290 \end{array}\right]$
Conv + TFM (ours)	SpCaNet-1	224^2^	18.2	3336.8	99.18	99.32	98.99	99.15	0.99	$\left[\begin{array}{cc} 306 & 6\\ 3 & 293 \end{array}\right]$
	SpCaNet-2	224^2^	33.6	6846.4	98.52	97.99	98.99	98.49	0.99	$\left[\begin{array}{cc} 306 & 6\\ 3 & 293 \end{array}\right]$
	SpCaNet-3	224^2^	56.1	12883.4	99.34	98.67	100	99.33	0.99	$\left[\begin{array}{cc} 308 & 4\\ 0 & 296 \end{array}\right]$
	SpCaNet-4	224^2^	118	27737.1	99.18	100	98.31	99.15	0.99	$\left[\begin{array}{cc} 312 & 0\\ 5 & 291 \end{array}\right]$
	SpCaNet-5	224^2^	205	48607.2	99.84	99.66	100	99.83	0.99	$\left[\begin{array}{cc} 311 & 1\\ 0 & 296 \end{array}\right]$

**Table 8:** Comparison of GAM and SGD.

Model	Number of total epochs	GAM (%)	SGD (Without GAM) (%)
efficientnetv2_l (Tan and Le, 2019)	50	98.84(0.4 ± 0.28)	98.68(0.8 ± 0.31)
	200	98.96(0.6 ± 0.68)	98.71(0.8 ± 0.73)
ResNet152 (He, et al., 2016)	50	98.23(0.6 ± 0.52)	98.85(0.4 ± 0.58)
	200	98.84(0.5 ± 0.33)	98.72(0.9 ± 0.82)
DeiT_B (H. Touvron, et al, “Training data-efficient image transformers distillation through attention,” International Conference on Machine Learning. PMLR, 2021)	50	96.84(0.7 ± 0.62)	95.23(2.6 ± 0.47)
	200	97.24(0.9 ± 0.76)	96.10(2.2 ± 0.17)
ViT-B/16 (B. L. Dosovitskiy A, Kolesnikov A, et al, “An image is worth 16x16 words: Transformers for image recognition at scale[J],” arXiv preprint arXiv:, et al., 2010)	50	89.72(2.9 ± 0.96)	87.17(3.1 ± 0.56)
	200	90.30(2.2 ± 0.68)	89.15(2.1 ± 1.04)
ViT-L/16 (B. L. Dosovitskiy A, Kolesnikov A, et al, “An image is worth 16x16 words: Transformers for image recognition at scale[J],” arXiv preprint arXiv:, et al., 2010)	50	95.67(1.9 ± 0.79	96.22(1.9 ± 0.77)
	200	96.14(1.1 ± 1.25)	94.61(2.9 ± 1.05)
Swin-B (Liu, et al., 2021)	50	97.24(0.9 ± 0.71)	96.38(2.4 ± 0.35)
	200	96.45(0.8 ± 0.77)	97.84(1.3 ± 0.94)
Swin-L (Liu, et al., 2021)	50	99.21(0.1 ± 0.52)	99.18(0.1 ± 0.27)
	200	98.84(0.4 ± 0.94)	98.72(0.8 ± 0.51)
BotNet (Srinivas, et al., 2021)	50	98.93(0.2 ± 0.49)	97.53(2.0 ± 0.37)
	200	98.73(0.5 ± 0.68)	97.64(0.7 ± 0.91)
SpCaNet	50	99.48(0.1 ± 0.63)	99.18(0.3 ± 0.26)
	200	99.64(0.2 ± 0.37)	99.42(0.3 ± 0.39)

**Table 9:** Per-class classification results.

Class	Specificity	Precision	Recall	F1 Score
Benign	99.66	99.68	100.00	99.84
Malignant	100.00	100.00	99.66	99.83

**Table 10:** Statistical analysis of the accuracies.

Fold	Accuracy
1	99.67
2	99.18
3	99.51
4	99.34
5	98.68
Mean + SD	99.28 ± 0.34

**Table 11:** Comparison of the different methods.

Method	Accuracy (%)	Specificity (%)	Recall (%)	Precision (%)
SE-ResNeXt-MLT (C. N. Linqi J, Jingyang L,, 2022)	98.99	98.33	99.18	99.51
FCNN-CRFs (A. S. Gull S, Khan H U,, 2021)	97.31	95.83	98.14	97.69
KSVM-SSD (K. K. Rao C S,, 2022)	98.84	100.00	97.06	100.00
Ensemble (Breiman, 2017)	97	98	96	97
**GAM-SpCaNet (Ours)**	**99.28**	**100.00**	**99.66**	**100.00**

**Table 12:** The analyzed Results of SpCaNet by independent sample *t*-test.

Indicator	VarName	Sample size	mean	std	t	P	mean gap	Cohen's d
Accuracy	SOTA models	16	98.358	0.809	−3.184	0.003	0.711	1.126
	SpCaNets	16	99.069	0.377				
	Total	32	98.713	0.718				
Precision	SOTA models	16	97.65	1.128	−2.078	0.046	0.664	0.735
	SpCaNets	16	98.314	0.6				
	Total	32	97.982	0.951				

**Table 13:** The analyzed Results of GAM by independent sample *t*-test.

Indicator	VarName	Sample size	mean	std	t	P	mean gap	Cohen's d
Accuracy of 50th epoch	SGD	45	95.696	3.7	−2.007	0.048	1.433	0.423
	GAM	45	97.129	3.044				
	Total	90	96.412	3.445				
Accuracy of 200th epoch	SGD	45	95.941	3.257	−2.115	0.037	1.341	0.446
	GAM	45	97.282	2.735				
	Total	90	96.612	3.066				
